# Hereditary Hemochromatosis *(HFE) *genotypes in heart failure: Relation to etiology and prognosis

**DOI:** 10.1186/1471-2350-11-117

**Published:** 2010-07-29

**Authors:** Daniel V Møller, Redi Pecini, Finn Gustafsson, Christian Hassager, Paula Hedley, Cathrine Jespersgaard, Christian Torp-Pedersen, Michael Christiansen, Lars V Køber

**Affiliations:** 1 Department of Cardiology, Rigshospitalet, Copenhagen University Hospital, Copenhagen, Denmark; 2 Department of Clinical Biochemistry and Immunology, Statens Serum Institut, Copenhagen, Denmark; 3 Department of Cardiology, Gentofte Hospital, University of Copenhagen, Copenhagen, Denmark

## Abstract

**Background:**

It is believed that hereditary hemochromatosis (HH) might play a role in cardiac disease (heart failure (HF) and ischemia). Mutations within several genes are HH-associated, the most common being the *HFE *gene. In a large cohort of HF patients, we sought to determine the etiological role and the prognostic significance of *HFE *genotypes.

**Methods:**

We studied 667 HF patients (72.7% men) with depressed systolic function, enrolled in a multicentre trial with a follow-up period of up to 5 years. All were genotyped for the known *HFE *variants C282Y, H63D and S65C.

**Results:**

The genotype and allele frequencies in the HF group were similar to the frequencies determined in the general Danish population. In multivariable analysis mortality was not predicted by C282Y-carrier status (HR 1.2, 95% CI: 0.8-1.7); H63D-carrier status (HR 1.0, 95% CI: 0.7-1.3); nor S65C-carrier status (HR 1.2, 95% CI: 0.7-2.0). We identified 27 (4.1%) homozygous or compound heterozygous carriers of *HFE *variants. None of these carriers had a clinical presentation suggesting hemochromatosis, but hemoglobin and ferritin levels were higher than in the rest of the cohort. Furthermore, a trend towards reduced mortality was seen in this group in univariate analyses (HR 0.4, 95% CI: 0.2-0.9, p = 0.03), but not in multivariate (HR 0.5, 95% CI: 0.2-1.2).

**Conclusion:**

*HFE *genotypes do not seem to be a significant contributor to the etiology of heart failure in Denmark. *HFE *variants do not affect mortality in HF.

## Background

Hereditary hemochromatosis (HH), an autosomal recessive disease with incomplete penetrance and variable expressivity, leads to progressive iron accumulation and deposition in several organs including liver, pancreas and in severe, untreated cases the heart. Clinically, HH manifests as increased erythropoiesis, liver cirrhosis, diabetes mellitus, hepatocellular carcinoma and potentially heart disease (heart failure, ischemia and arrhythmia, especially in juvenile hemochromatosis) [1,2]. Mutations within several genes have been associated with HH, these include hemochromatosis (*HFE*), hemojuvelin (*HJV*)[3], transferrin receptor 2 (*TfR2*)[4], and hepcidin (*HAMP*)[5]. In 1996 the *HFE *gene was identified and the C282Y and H63D polymorphisms associated with HH[6]. Most HH-patients in Northern Europe are C282Y homozygotes, and these account for 80-90% of the HH cases, with compound heterozygotes (where an individual has two different abnormal alleles at a particular locus) being responsible for an additional few percent[7]. The biochemical prevalence of HH in Denmark has been estimated to 0.37-0.46%[8] and 94.8% of diagnosed HH-patients are homozygous for the *HFE *C282Y polymorphism[9].

Iron plays a variety of roles in cellular function and variations in iron content could play a pathophysiological role in several heart diseases due to interference in oxidation-reduction reactions and cellular proliferation[10]. *HFE *polymorphisms have been associated to the occurrence of ischaemic heart disease[11], but this association is controversial and has not been confirmed in a large meta-analysis[12]. Some studies concerning idiopathic dilated cardiomyopathy and *HFE *polymorphisms have been unable to demonstrate a clear association[13,14], whereas another, focusing on cardiomyopathies of different etiologies, found that polymorphisms in the *HFE *gene was associated with ischemic heart disease, but there were neither biochemical markers nor survival data available[15].

We hypothesized that *HFE *genotypes might play an etiological role in heart failure (HF) or that variants in *HFE *might modify the clinical prognosis of HF.

We explored this hypothesis in two ways. Firstly, we examined the frequency of the most common *HFE *polymorphisms C282Y (dbSNP rs 1800562), H63D (dbSNP rs 1799945) and S65C (dbSNP rs 1800730) in a large HF population and assessed whether the *HFE *genotypes C282Y/C282Y, H63D/H63D, S65C/S65C, C282Y/H63D, C282Y/S65C and H63D/S65C, causing HH, were overrepresented in HF, suggesting an etiological role. Secondly, we studied the relation between different *HFE *genotypes and all-cause mortality in the same population.

## Methods

### Subjects and clinical investigations

The study population was based on consecutive patients enrolled in the Echocardiography and Heart Outcome Study (ECHOS). ECHOS was a consecutive, prospective, double-blind, randomized, placebo-controlled Scandinavian multicentre trial. It evaluated the effect of a selective agonist of the pre-synaptic DA_2_-dopaminergic and α_2_-adrenergic receptors in patients with moderate to severe heart failure of any etiology, with New York Heart Association (NYHA) functional class II-IV. The study was neutral with respect to every preselected endpoint and biological measure[16]. To be eligible for inclusion in the study, patients were required to have a history of dyspnoea or fatigue at rest or minimal exertion, corresponding to NYHA class III-IV within the last month, requiring treatment with diuretic and had to be in NYHA class II-IV at time of randomization. Furthermore, an echocardiogram recorded at the local hospital was evaluated in a core lab prior to randomization. To be included, depressed left ventricular function corresponding to wall motion index (WMI) ≤ 1.2 (using a reverse scoring system; corresponding to a left ventricular ejection fraction ≤ 35%) had to be present[17]. Patients with acute coronary syndrome were not eligible for the study nor were patients with atrioventricular block grade II or III, clinically significant hepatic (severe cirrhosis) or renal disease (serum creatinine > 300 μmol/l), stroke within 1 month, or any illness or disorder other than heart failure which could preclude participation or severely limit survival. The local investigators classified the patients in different groups according to etiology as described previously[18]. The study conforms to the principles outlined in the Declaration of Helsinki and was approved by The Danish Board of Health as well as the Central Danish Ethics Committee. All patients gave written informed consent. There were 1000 patients randomized in the study. To ensure long term mortality status, only Danish patients with available blood samples were included in the present study, leaving 667 patients available for genotyping and follow up. Survival status was obtained from the Danish Central Person Register, where all deaths are registered within 2 weeks. The register was interrogated in July 2006, resulting in a follow up period of up to 5 years. There were no *HFE *genotyped patients lost to follow up.

Demographic data, NYHA class, left ventricular ejection fraction, medical history and medication at admission and at discharge as well as in-hospital complications were recorded for all randomized patients.

As the randomized study was neutral with regard to every preselected endpoint and biological measure, we made the present analysis without making any distinction between the two study treatment groups.

### Molecular genetic studies

Genomic DNA was isolated from whole blood samples using the Maxwell^®^16 system (Promega, USA). The regions of *HFE *(Genbank accession no. NM_139011) containing C282, H63 and S65 were amplified by polymerase chain reaction (primers available upon request). Subsequently genotyping was performed by multiplex pyrosequencing using the Pyro Gold Reagents kit (Biotage, Uppsala, Sweden) and processed in a PSQ 96MA (Biotage, Uppsala, Sweden).

### Statistical analyses

Continuous variables are summarized as medians with 5^th ^and 95^th ^percentiles and group comparisons performed by Wilcoxon rank sum test. Categorical variables are expressed as number and percentage of the total group and comparative analysis was done using the χ^2^-test. Survival and event rates were determined with the Kaplan-Meier method and comparisons between groups were performed with the log rank test. Multivariable analysis was performed using Cox proportional hazard models. All variables, except medication, were included in the multivariable analyses. In the Cox proportional hazard model, BNP was logarithmic transformed. Each *HFE *variant was dichotomized into carriers (hetero- and homozygote carriers) and non-carriers due to small sample size. We also evaluated the variables "any variant" and "HH-genotype"; comparing any HFE variant combination or genotypes associated to HH (C282Y/C282Y, H63D/H63D, C282Y/H63D, C282Y/S65C and H63D/S65C) to wild type of all three variants, respectively. Hardy-Weinberg equilibrium was tested by a χ^2^-test with 6 degrees of freedom.

Data were analyzed using the Statistical Analysis Software (SAS version 9.1). Results were considered significant at *p*-value < 0.05.

## Results

Clinical baseline characteristics of the 667 patients and their *HFE *variant status are summarized in table 1. The hemoglobin level in H63D carriers and the creatinine level in S65C and "any variant"-carriers were higher compared to non-carriers (p = 0.013; 0.025 and 0.04, respectively). There were a lower frequency of patients with a history of ischemic heart disease in the C282Y carrier group as well as in the "any variant" carrier group (p = 0.048 and 0.009). Otherwise there was no statistically difference with respect to any of the variables between carriers and non-carriers.

**Table 1:** Clinical baseline characteristics of 667 patients hospitalized with symptomatic heart failure according to *HFE *genotype

HFE variant	C282Y		H63D		S65C		Any variation	
**Status**	Carrier	non-carrier	Carrier	non-carrier	Carrier	non-carrier	Carrier	Non-carrier

n total	73	594	144	523	26	641	231	436

**Clinical data**								

Age (years)	72.0 (43.6-86.8)	71.1 (49.5-85.9)	71.3 (46.5-87.8)	71.1 (49.5-85.3)	72.6 (46.0-80.6)	71.1 (48.4-86.0)	71.5 (46.0-87.1)	71.0 (50.0-85.9)

male	55 (75)	430 (72)	106 (74)	379 (72)	18 (69)	467 (73)	170 (74)	315 (72)

BMI (kg/m^2^)	26.4 (20.3-35.1)	26.0 (18.8-35.3)	26.4 (18.7-34.4)	25.9 (19.2-35.3)	26.0 (16.7-44.7)	26.0 (19.1-35.1)	26.3 (19.2-35.1)	25.8 (19.1-35.3)

Current smoker (%)	21 (29)	191 (33)	41 (29)	171 (33)	11 (44)	201 (32)	69 (30)	143 (33)

NYHA classes (%)								

NYHA class I	1 (1)	21 (3)	7 (5)	10 (2)	0	17 (3)	8 (3)	9 (2)

NYHA class II	12 (16)	181 (28)	42 (29)	122 (23)	6 (23)	158 (25)	58 (25)	106 (24)

NYHA class III	47 (64)	360 (56)	76 (53)	315 (61)	15 (58)	376 (59)	132 (57)	259 (60)

NYHA class IV	13 (18)	80 (12)	19 (13)	73 (14)	5 (19)	87 (14)	33 (14)	59 (14)

**Medical history**								

CHF (%)	62 (86)	502 (85)	115 (80)	449 (86)	23 (86)	541 (85)	189 (83)	375 (86)

IHD (%)	30 (41)*	317 (53)	65 (45)	282 (54)	14 (54)	333 (52)	104 (45)¤	243 (56)

Former AMI (%)	23 (32)	217 (37)	51 (35)	189 (36)	9 (36)	231 (36)	78 (34)	162 (37)

Hypertension (%)	24 (33)	139 (23)	36 (25)	127 (24)	9 (35)	154 (24)	66 (29)	97 (22)

COPD (%)	14 (19)	127 (21)	31 (22)	110 (21)	8 (31)	133 (21)	52 (23)	89 (20)

Diabetes diagnosis (%)	13 (18)	102 (17)	25 (17)	90 (17)	7 (27)	108 (17)	42 (18)	73 (17)

Hyperlipidaemia (%)	22 (32)	217 (37)	50 (35)	189 (37)	9 (36)	230 (37)	76 (34)	163 (38)

Stroke/TCI (%)	14 (19)	70 (12)	16 (11)	68 (13)	6 (23)	78 (12)	34 (15)	50 (11)

**Medication at discharge**								

ACE-I (%)	52 (72)	474 (80)	115 (80)	411 (79)	20 (77)	506 (79)	178 (77)	348 (80)

AT-II blockers (%)	7 (10)	53 (9)	13 (9)	47 (9)	4 (15)	56 (9)	24 (10)	36 (8)

β-Blokage (%)	43 (60)	298 (50)	70 (49)	271 (52)	14 (54)	327 (51)	121 (53)	220 (51)

Diuretics (%)	71 (99)	578 (98)	142 (99)	507 (98)	25 (96)	624 (98)	226 (98)	423 (98)

**Paraclinical data**								

WMI	1.0 (0.4-1.2)	0.9 (0.5-1.2)	0.9 (0.5-1.2)	0.9 (0.4-1.2)	0.9 (0.4-1.2)	0.9 (0.5-1.2)	0.9 (0.4-1.2)	0.9 (0.5-1.2)

Serum Ferritin (ng/ml)	121 (31-582)	123 (35-430)	129 (29-430)	122 (36-443)	159 (42-260)	121 (35-461)	127 (29-417)	121 (37-466)

Hemoglobin (mmol/l)	8.8 (7.1-10.8)	8.6 (6.7-10.4)	8.7 (7.0-10.4)†	8.6 (6.7-10.4)	8.4 (6.9-9.6)	8.6 (6.8-10.4)	8.7 (7.0-10.4)¶	8.5 (6.6-10.4)

Serum creatinin (μmol/l)	99 (68-259)	106 (68-203)	108 (70-201)	104 (67-208)	120 (73-251)‡	105 (68-198)	108 (68-222)	103 (67-194)

BNP (pmol/L)	1.5 (0.3-4.0)	1.3 (0.4-4.1)	1.3 (0.3- 3.7)	1.4 (0.4- 4.1)	1.3 (0.2- 4.7)	1.3 (0.4-4.0)	1.3 (0.3-4.2)	1.3 (0.4-4.0)

BMI = Body Mass Index; NYHA = New York Heart Association functional class; HF = Heart Failure; IHD = Ischemic Heart Disease; AMI = Acute Myocardial Infarction; COPD = Chronic Obstructive Pulmonary Disease; TCI = Transient Cerebral Ischemia; ACE-I = Angiotensin Converting Enzyme-Inhibitor; AT-II = Angiotensin II receptor; WMI = Wall Motion Index; BNP = Brain Natriuretic Peptide; * p = 0.046; † p = 0.007; ‡ p = 0.025; ¤ p = 0.002; ¶ p = 0.022

The *HFE *genotype distribution is displayed in table 2 in conjunction with a newly published result obtained in a large Danish general population cohort[19] and is consistent with the Hardy-Weinberg equilibrium (χ^2 ^= 1.97, p = 0.922). No significant differences were found between the two populations.

**Table 2:** Distribution of *HFE *genotypes in 667 heart failure patients, compared to frequencies found among 6020 Danish men (Pedersen et al.[19]) showing no differences between the two studies

*Genotype*			*Distribution*			***Pedersen et al***[19]
C282Y	H63D	S65C	n	n expected*	(%) of total†	(%)/n

C/C	H/H	S/S	436	434	65.4	64.3/3871

Y/C	H/H	S/S	61	61	9.2	8.4/503

Y/C	D/H	S/S	8	9	1.2	1.4/85

Y/C	H/H	C/S	1	2	0.2	0.1/9

Y/Y	H/H	S/S	3	2	0.4	0.4/23

C/C	D/H	S/S	121	126	18.1	20.1/1208

C/C	D/H	C/S	3	3	0.4	0.4/27

C/C	D/D	S/S	12	9	1.8	1.8/110

C/C	H/H	C/S	22	21	3.3	3.0/183

C/C	H/H	C/C	0	0	0	0/1

Total			667	667	100	100/6020

* according to Hardy-Weinberg equilibrium; † of observed

We found 231 (34.6%) of the patients to carry at least one of the three *HFE *polymorphisms. The allele frequencies of *HFE *polymorphisms C282Y, H63D and S65C were 5.7%, 11.7% and 1.9%, respectively. We found homozygosity in three patients for C282Y, in 12 for H63D and none for S65C. Compound heterozygosity was present in 12 patients. Thus, 27 patients had a genotype suggestive of HH. None of the homozygotes had markedly elevated serum ferritin (all < 1000 ng/ml), while one compound heterozygote (C282Y/H63D) had markedly elevated serum ferritin level (1528 ng/ml). Median ferritin level was significantly higher among the 27 patients, compared to non-carriers (p = 0.021). None had a history of HH, nor developed it during the follow up period.

The homozygotes and compound heterozygotes had a significantly lower mortality (p = 0.019) when compared with the rest of the patients in univariate analyses (HR 0.4, 95% CI: 0.2-0.9). The significance diminished in multivariable analyses, leaving it insignificant (HR 0.5, 95% CI: 0.2-1.2). The genotypes associated with HH had significantly higher levels of serum ferritin, but not to an extent believed to cause organ damage. In the three C282Y homozygotes, which carry the presumably highest risk of developing HH, we examined hospital records to explore the possibility of a missed HH-diagnosis. Ferritin levels were normal (< 300 ng/ml) in all three patients (two men). One suffered from diabetes, while two had cerebral stroke as well as atrial fibrillation. Two of them had died, at the ages 80 and 98 years, respectively. The last one, a male age 80 years old is still alive. None of them had liver involvement or a history of skin pigmentation. There were no clinically indices supporting the HH diagnosis. However, no histological material was available to exclude iron deposition.

Table 3 summarizes the distribution of the etiologies of HF (where known) as a function of carrier or non-carrier status for each polymorphism. No association between polymorphisms and etiology was found. The relation between variant carrier status and mortality during the follow-up period (median 51 months (range: 33.6 - 64.6 months, 328 deaths (49.2%)) is also given and the S65C polymorphism is associated with significantly (p = 0.017) increased mortality. Univariate Cox proportional hazards analyses was performed to assess the impact of different *HFE *genotypes and their carrier-status on all-cause mortality. Only the S65C carrier status was a significant predictor of all-cause mortality (HR 1.9, 95% CI: 1.2-3.0). In multivariable Cox proportional hazard analyses the impact on all-cause mortality decreased, leaving it insignificant (HR 1.2, 95% CI: 0.7-2.0). Serum ferritin was not associated with mortality (HR 1.00; 95% CI: 0.99-1.01, p = 0.88). Significant predictors were: age (HR 1.03, 95% CI: 1.02-1.04); diabetes (HR 1.31, 95% CI: 1.00-1.75); history of stroke (HR 1.39, 95% CI: 1.01-1.91); hemoglobin-level (HR 0.88, 95% CI: 0.78-1,00); BNP (HR 1.38, 95% CI: 1.21-1.58) and chronic obstructive pulmonary disorder (HR 2,08, 95% CI: 1.60-2.70).

**Table 3:** *HFE *genotype distribution according to etiology and mortality

Variant	Genotype	Distribution	IHD (n = 332)	Hypertension (n = 61)	DCM (n = 108)	Valve disease (n = 42)	Other (n = 57)	Unknown (n = 67)	Deceased (n = 328) (%)
C282Y	Non-carrier	594	303 (51.0)	51 (8.6)	93 (15.7)	38 (6.4)	49 (8.3)	60 (10.1)	290 (48.8)
	
	Carrier	73	29 (39.7)	10 (13.7)	15 (20.6)	4 (5.5)	8 (11.0)	7 (9.6)	38 (52.0)

H63D	Non-carrier	523	268 (51.2)	46 (8.8)	80 (15.3)	35 (6.7)	39 (7.5)	55 (10.5)	263 (50.3)
	
	Carrier	144	64 (44.4)	15 (10.4)	28 (19.4)	7 (4.9)	18 (12.5)	12 (8.3)	65 (45.1)

S65C	Non-carrier	641	318 (49.6)	59 (9.2)	104 (16.2)	41 (6.4)	54 (8.4)	65 (10.1)	309 (48.2)
	
	Carrier	26	14 (53.9)	2 (7.7)	4 (15.4)	1 (3.9)	3 (11.5)	2 (7.7)	19 (73.1)*

Any	Non-carrier	436	230 (52.8)	35 (8.0)	65 (14.9)	30 (6.9)	30 (6.9)	46 (10.6)	210 (48.2)
	
	Carrier	231	102 (44.2)	26 (11.3)	43 (18.6)	12 (5.2)	27 (11.7)	21 (9.1)	118 (51.1)

DCM = dilated cardiomyopathy, IHD = ischemic heart disease, Other = HF due to atrial fibrillation or rare diseases, Unknown = no single disease could be assigned as the cause of HF, Deceased = deceased in the follow up period, *p = 0.019

## Discussion

The present study demonstrates in a large population hospitalized for symptomatic heart failure that the distribution of *HFE *polymorphisms C282Y, H65D and S63C is not dissimilar from the distribution found in the general Danish male population and that *HFE *genotypes is not a significant etiological factor in heart failure. None of the *HFE *polymorphisms carried independent prognostic information, but homozygotes or compound heterozygotes, despite having increased risk of HH, had a trend towards reduced mortality.

The hemochromatosis gene encodes *HFE*, a transmembrane glycoprotein which normally associates with transferrin receptor 1 (TfR1) and decreases intracellular iron and ferritin concentrations. New insights into the function of the *HFE *protein suggests an iron sensing function, as well as a hepcidin regulating function[20] and subsequently a pivotal role in iron homeostasis. The C282Y polymorphism disrupts the TfR1 association leading to accumulation of intracellular iron and loss of iron homeostasis. How the two other polymorphisms H63D and S65C influence iron homeostasis has not yet been clarified, as they do not interrupt the TfR1 association. All three polymorphisms are associated with HH[21] with variable disease expression and penetrance. The *HFE *H63D polymorphism also associates with neurodegenerative disorders[22] and cerebral stroke[23] among others. We confirm the lack of association between *HFE *polymorphisms and IHD in our material, as well as confirming the lack of association with dilated cardiomyopathy. In addition, we found no association between the remaining etiologies to heart failure and the frequencies of the *HFE *polymorphisms.

Hereditary hemochromatosis has been described as a potential cause of heart failure and arrhythmias[24] and as a consequence we assessed a large heart failure population with different etiology using *HFE *genotyping and serum ferritin measurements. The distribution of *HFE *genotypes was similar to prior Danish studies[19] indicating that the role of HH appears low. The well known variable biochemical and clinical expressivity of the homozygote C282Y polymorphism can explain the discrepancy found between the frequency of C282Y homozygotes in the general population and HH[25]. The concurrence of genetic and nongenetic factors in the development of HH due to *HFE *polymorphisms seems present. Studies investigating modifiers of the penetrance in *HFE *related hemochromatosis has been conflicting in humans[26,27] despite convincing animal models[28].

To our knowledge, this is the first study that examines *HFE*-polymorphisms and their association with all-cause mortality in a heart failure population. Dunn et al[29] found no association between *HFE *polymorphisms and cardio-vascular disease mortality in patients with coronary artery disease. In our study we found no association between *HFE *genotype and all-cause mortality irrespective of heart failure etiology including ischemic heart disease and dilated cardiomyopathy. Although we did not specify the cause of death to be cardiac or non-cardiac related as the study by nature is observational, all patients suffered from severe heart failure (NYHA class III-IV episode within a month prior to inclusion) why the main cause of death should be expected to be due to cardiovascular disorders. This fits well with prior investigations performed where cardiac mortality rates of about 50% over a 5-year period in HF-patients is seen[30]. The S65C polymorphism seemed to significantly increase mortality when tested in univariate analyses and keeping in mind the low number in this group it lost its significance when applying available covariates.

The significantly higher hemoglobin level seen in the "HH-genotype" group could explain the tendency towards decreased mortality seen in the group. It is well established that anemia in heart failure increases mortality[31] It could be speculated that the *HFE *genotypes predisposing HH, in our population represents a mild clinical form, not leading to organ damage but only altering hepcidin levels leading to increased erythropoiesis. Univariate analyses were significant, but due to the small sample size (27 patients) especially with the C282Y homozygous polymorphism (3 patients) we did not have enough statistical power to make any definite conclusions.

Patients with severe liver affection, i.e. biochemical suspicion of cirrhosis of the liver, were excluded from the study ruling out the presence of overt classical HH. Subsequently, we investigated the role of the various *HFE *genotypes. A possible source of bias could be the exclusion of patients with cerebral stroke within the last month as Ellervik et al[23] found a positive association between *HFE *genotypes and stroke. Thereby we could have lost patients, with a *HFE *genotype but without overt HH, although the amount of patients missed must be negligible, given the low frequency of HH. Furthermore, none of the excluded patients was diagnosed with HH during the follow up period assessed by the Danish Patient Register.

## Conclusion

In conclusion, *HFE *genotypes were not associated with systolic heart failure, irrespective of etiology. Secondly, *HFE *genotypes did not significantly affect prognosis. Therefore, it seems that performing *HFE *genotype screening in heart failure patients has no value, unless they display clear clinical signs of HH, including biochemical markers as elevated serum ferritin, transferrin saturation and in the future possibly also decreased hepcidin levels. Future studies should evaluate if certain *HFE *genotypes could have a modifying effect on heart failure mortality due to the effect on hepcidin and subsequently hemoglobin levels.

## Competing interests

The authors declare that they have no competing interests.

## Authors' contributions

DVM, MC and LK did the conception and design of the study as well as interpreting the data. CH, LK and CTP did the conception and design as well as interpreting the data from the ECHOS trial. RP and FG analyzed and interpreted the statistical data. PH and CJ performed the molecular genetic studies and their interpretation. All authors contributed to the critical revision of the paper and have read and agreed to the manuscript as written.

## Pre-publication history

The pre-publication history for this paper can be accessed here:

http://www.biomedcentral.com/1471-2350/11/117/prepub
